# Three-Dimensionally Printed Bionic Hydroxyapatite (HAp) Ceramic Scaffolds with Different Structures and Porosities: Strength, Biocompatibility, and Biomedical Application Potential

**DOI:** 10.3390/ma17246092

**Published:** 2024-12-13

**Authors:** Peng Zhang, Qing Zhou, Rujie He

**Affiliations:** 1 School of Management, Beijing Institute of Technology, Beijing 100081, China; pengzhang@bit.edu.cn; 2 Institute of Advanced Structure Technology, Beijing Institute of Technology, Beijing 100081, China; zhouqing@iast.bit.edu.cn

**Keywords:** hydroxyapatite, scaffold, 3D printing, mechanical properties, biocompatibility

## Abstract

Bionic bioceramic scaffolds are essential for achieving excellent implant properties and biocompatible behavior. In this study, inspired by the microstructure of natural bone, bionic hydroxyapatite (HAp) ceramic scaffolds with different structures (body-centered cubic (BCC), face-centered cubic (FCC), and gyroid Triply Periodic Minimal Surfaces (TPMSs)) and porosities (80 vol.%, 60 vol.%, and 40 vol.%) were designed, 3D-printed, and characterized. The effects of structure and porosity on the morphology, mechanical properties, and in vitro biocompatibility properties of the HAp scaffolds were studied and compared with each other. Interestingly, the HAp scaffold with a porosity of 80 vol.% and a TPMS structure had the best combination of compressive strength and in vitro biocompatibility, and demonstrated a great biomedical application potential for bone repair. We hope this study can provide a reference for the application and development of HAp scaffolds in the field of bone repair engineering.

## 1. Introduction

The risks of osteoporosis and bone defects caused by accidental trauma and congenital diseases have always been significant [1,2]. Autologous bone grafting is one of the important methods in reconstructive surgery, but the availability of autologous bone is limited, and a second surgery is needed during its collection process, easily leading to severe complications. Currently, researchers and clinicians are seeking bionic porous bone repair scaffolds as alternatives to autologous bone grafting [3,4,5,6]. Extensive research has been conducted on bone repair scaffolds made of biometals [7], biopolymers [8], bioceramics [9,10], and biocomposites [11,12]. Among them, bioceramics have excellent biosafety and tissue compatibility with bone tissue, making them the most attractive option for bone repair materials.

Among various bioceramics, hydroxyapatite (HAp) has good biocompatibility and osteoinductivity [13,14,15]. It is more crystalline than natural bone minerals, making the implant chemically more stable and less susceptible to degradation upon implantation. Moreover, HAp is widely distributed in animal bone tissue and is a major component of natural bone, accounting for up to 70% of human bone. Therefore, HAp exhibits good biocompatibility, slow in situ biodegradation, and excellent osteoconductivity and osteoinductivity [16,17,18,19,20]. After HAp scaffolds are implanted, new bone can grow through the pores of the scaffolds and connect with the surrounding bone tissue at the defect site. Liu et al. [21] evaluated the in vitro biocompatibility and degradability of HAp through synthetic body fluid immersion tests and MTT cell proliferation experiments. They found no significant dissolution or cytotoxicity, verifying its biostability and biosafety. The in vivo osteogenic capacity of HAp has also been reported. Chen et al. [22] implanted HAp scaffolds into rabbits to observe the osteogenic effect. The results showed no adverse physiological reactions such as infection in the rabbits, and the HAp implants formed strong bone connections with the defects. In summary, HAp ceramic scaffolds have been widely used in the biomedical field, including as controlled drug release matrices and implantable materials for bone tissue engineering.

Particularly importantly, the ideal structure of artificial bone scaffolds should be similar to natural bone [5,6,18,19,20]. Therefore, the geometric appearance and pore structure of the scaffolds, including pore size, pore shape, pore orientation, porosity, connectivity, and the combination of pore morphologies at different locations, must also match the substitutes. Faced with this differentiation, traditional machining methods, such as material reduction manufacturing and molding, have many disadvantages, such as complex preparation processes, a lack of flexibility, high costs, and the inability to precisely control pore size, shape, distribution, and connectivity in the templating, pore-forming, and foaming processes, as well as the need to use toxic organic solvents. Additive manufacturing (3D printing) technology has the advantage of flexible design and can prepare various structures from micro- to macro-scales, more accurately adjusting and controlling the size and distribution of material pores and greatly reducing processing difficulty [16,17]. Currently, 3D printing usually contains the following techniques: material jetting (MJ), binder jetting (BJ), vat photopolymerization (VPP), material extrusion (ME), powder bed fusion (PBF), directed energy deposition (DED), sheet lamination (SL) [23]. Among them, vat photopolymerization has the advantages of high precision and fast preparation cycles, and has received increasing research attention in the 3D printing of bioceramic implants [16,17]. Chen et al. [23] prepared porous HAp scaffolds with a honeycomb structure through stereolithography-based VPP, with a hexagonal cross-section and an inscribed circle of 500 μm. After implanting the HAp structure into rabbits, micro-computed tomography (micro-CT) images showed no adverse physiological reactions such as infection in the rabbits, and a strong bone connection was formed between the HAp and the defect, indicating good biosafety of the HAp scaffolds in vivo. Luo et al. [24] modified direct ink writing-based MJ to prepare bioceramic scaffolds with designed macropores and hollow channels in multiple directions. Their study successfully presented scaffolds with high porosity, as well as excellent mechanical strength and specific surface area. Micro-CT analysis after implantation at 4 and 12 weeks showed that the implantation of the scaffolds significantly stimulated new bone growth. Previously, we also prepared HAp scaffolds through digital light processing-based VPP 3D printing [25].

In summary, numerous studies have demonstrated that 3D printing has opened up promising technical pathways for the preparation of complex heterogeneous structures of HAp bioceramics and the enhancement of osteogenic properties [26,27]. However, the performance of implants largely depends on their structural design, which we do not yet have an in-depth understanding of. Zhang et al. [28] investigated the mechanical properties of mandibular implants by using finite element analysis (FEA), and they finally ultimately that porous implants had higher strain, which was due to the uniform stress distribution caused by the presence of pores and the prevention of stress concentration compared to non-porous implants. Due to the relatively low mechanical properties of scaffold materials, such as trabecular bone (~12 MPa) or cortical bone (~200 MPa), the compressive strength of a pure hydroxyapatite porous blocks is ~0.3 MPa. Therefore, porous scaffolds with sufficient mechanical properties are necessary. Various structural designs have been reported, mainly focusing on grid structures, especially classic lattice structures with regular pore sizes, including BCC and FCC structures [16,17,29]. Parisien et al. [30] explored the mechanical performance of FCC and BCC structures and their potential to support bone induction in bone reconstruction applications, demonstrating that these structures could support bone growth and show great application potential for bone implants. Wang et al. [31] prepared calcium phosphate ceramics with an FCC structure and evaluated the mechanical behaviors and biological properties of the scaffolds. They found that the scaffolds effectively promoted the formation of new bone. Liang et al. [32] prepared HAp scaffolds with a BCC structure through VPP to assess their mechanical and biological properties. The results showed that the scaffolds had high compressive strength and good cell metabolism, indicating that lattice structures have the ability to support bone repair and reconstruction. In addition to lattice structures, a structure with periodic surfaces on the X, Y, and Z axes has received much attention in recent years due to its large specific surface area, uniform stress distribution, and trabecular bone-mimicking hyperbolic morphology, known as a Triply Periodic Minimal Surface (TPMSs). TPMS structures have near-zero surface curvature, avoiding stress shielding and improving stress concentration under force. There are various types of TPMS structures, commonly including Schoen Gyroid (G), Schoen IWP (I), and Schwarz Primitive (P) [33,34,35]. TPMS-based porous structures provide sufficient compressive strength for bone implants and bone regeneration. Zaharin et al. designed lattice and gyroid TPMS structures to evaluate their mechanical and biological properties, and it was found that gyroid TPMS had excellent mechanical properties. Bobbert et al. [36] prepared a titanium alloy with a TPMS structure through selective laser melting (SLM)-based 3D printing. This structure can largely simulate the mechanical properties and mass transport of bone, meeting the requirements of porous biomaterials. In our previous studies, we designed and 3D printed HAp scaffolds with different structures at a certain porosity of 40 vol.%, and investigated their morphologies and mechanical and in vitro behaviors [25]. These studies have shown that TPMS structures exhibit superior potential in supporting cell expansion, attachment, and stress shielding. However, although there have been some studies about bionic-structured HAp bioceramic scaffolds and their related properties, there has been no definitive conclusion on the comparison of mechanical and biological properties between FCC, BCC, and TPMS bioceramic structures. Finding the optimal structure of HAp bioceramic scaffolds, including their structural configuration and porosity, is deemed necessary for their use in biomedical applications.

Therefore, bionic hydroxyapatite (HAp) ceramics, which are the most promising for bone repair among bioceramics, were selected and combined with structure design in this study. Inspired by the microstructure of natural bone, bionic HAp ceramic scaffolds with different structures (body-centered cubic (BCC), face-centered cubic (FCC), and gyroid Triply Periodic Minimal Surfaces (TPMSs)) and porosities (80 vol.%, 60 vol.%, and 40 vol.%) were designed. The aim of this study is to find the optimal structure and porosity for HAp bioceramic scaffolds. In this study, the effects of structure and porosity on the morphology, compressive strength, and in vitro behaviors of the HAp scaffolds were studied and compared with each other. We want to provide a reference for the application and development of HAp scaffolds in the field of bone repair engineering.

## 2. Experimental Section

### 2.1. Bionic Design

**Figure 1:**
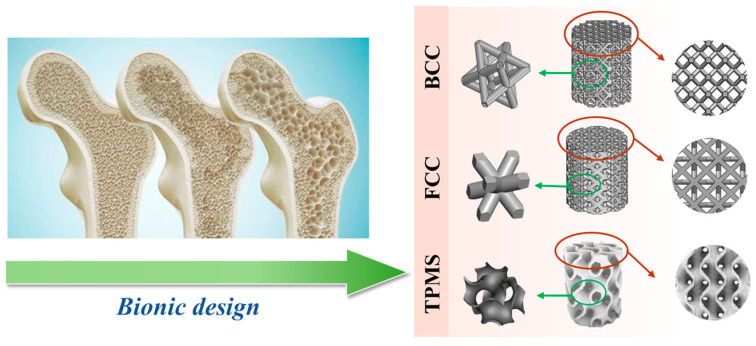
Bionic design of HAp scaffolds with different structures and porosities.

**Figure 2:**
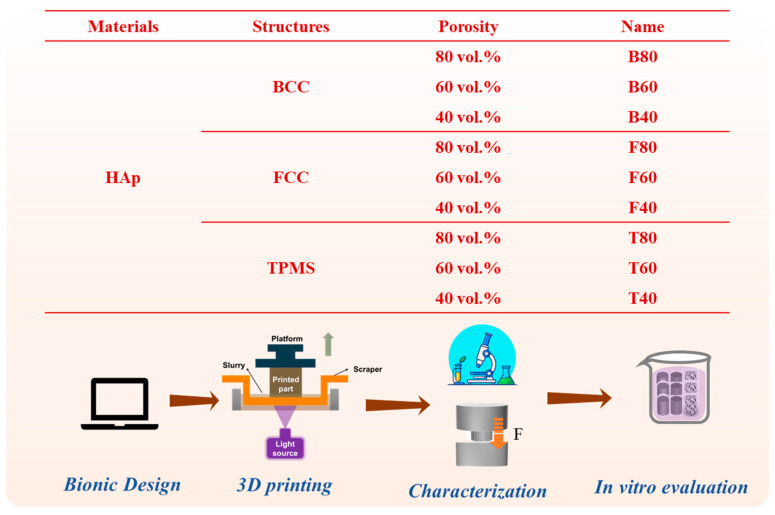
Design and flow chart of this study.

Figure 1 shows three different bionic ceramic scaffolds with various structures, including body-centered cubic (BCC), face-centered cubic (FCC), and gyroid (which is one typical type of TPMS). According to our previous works, a porosity of bioceramic scaffolds between 20 vol.% and 80 vol.% is suitable for bone tissue implants [17,21,25]. To the best of our knowledge, too high a porosity is not good for strength, and too low a porosity is not suitable for cell growth and biocompatibility [37,38]. Therefore, the porosity of the HAp scaffolds was set to 80 vol.%, 60 vol.%, and 40 vol.%, as given in Figure 2. In our bionic design, all the HAp bioceramic scaffolds had a diameter and a height of 8 mm and 8 mm, respectively. In this study, the bionic HAp scaffolds with different structures and porosities were named B80, B60, B40, F80, F60, and F40, and T80, T60, and T40, respectively, for simplicity.

### 2.2. Three-Dimensional Printing

#### 2.2.1. Raw Materials

The raw bioceramic material used in this study was commercial hydroxyapatite (HAp, purity > 98%) powder, which has an average diameter of 12 mm and was purchased from Nanjing Duly Biotech Co., Ltd., Nanjing, China. For 3D printing, 1,6-hexanediol diacrylate (HDDA, chemical pure) and trimethylolpropane triacrylate (TMPTA, chemical pure) were used as the light-curing resin monomers in this study. Both HDDA and TMPTA were purchased from Sinopharm Chemical Reagent Co., Ltd., Beijing, China. During the 3D printing process, free radical photoinitiator diphenyl(2,4,6-trimethyl-benzoyl) phosphine oxide (TPO, analytically pure), which was also provided by Sinopharm Chemical Reagent Co., Ltd., China, was used as a photoinitiator for vat photopolymerization. Homogeneous and dispersed HA slurry is very important for 3D printing; the dispersant used here was Solsperse KOS163, which was the chemical product ID, and was obtained from Guangzhou Qian’an Chemical Co., Ltd., Guangzhou, China.

#### 2.2.2. Three-Dimensional Printing

Figure 2 also presents a flow chart of this study. After their design, the bionic HAp scaffolds were VPP 3D printed. The whole 3D printing process consisted of three steps: slurry for 3D printing, VPP 3D printing, and sintering. Details of these three procedures are given below:

(1) Photosensitive slurry for 3D printing

Photosensitive ceramic–resin slurry was the basis for the subsequent VPP 3D printing. The resin monomers consisted of HDDA and TMPTA, with a HDDA/TMPTA volume ratio of 4:1. The ceramic powders, HAp powders, were weighed as a solid loading of 30 vol% in the ceramic–resin total volume. A TPO photoinitiator (2 wt% of the solid content) was added to the ceramic–resin slurry and ball-milled for 2 h at 400 rpm. 5 wt.% Solsperse KOS163, which was weighted based on the weight of the added HDDA, TMPTA, and HAp, was added as the dispersant. Subsequently, the slurry was ball-milled for another 24 h. Finally, photosensitive HAp ceramic–resin slurry was obtained for the subsequent VPP 3D printing.

(2) VPP 3D printing

VPP 3D printing of the HAp green body was conducted using commercial 3D printing equipment (AutoCera, Beijing 10dim Tech. Co., Ltd., Beijing, China). During VPP 3D printing, the thickness of each slice was 50 mm. The wavelength, intensity, and exposure time of the UV light were 405 nm, 9000 mW·cm^−^^2^, and 10 s, respectively. After the HAp slurry was cured layer by layer, the HAp green body was obtained.

(3) Sintering

After VPP 3D printing, the HAp green body was sintered in a Muffle furnace (Hefei Facerom Furnace Co., Ltd., Hefei, China). The whole sintering process was carried out in an air atmosphere. Firstly, the 3D-printed HAp green body was debinded to 650 °C at 1 °C·min^−^^1^ and held for 2 h. Then, the scaffold was further sintered at 1250 °C at 1 °C·min^−^^1^ and held for 2 h. Finally, the sample was cooled to room temperature at 1 °C·min^−^^1^.

### 2.3. Characterization

#### 2.3.1. Morphological Observation

The morphologies of the 3D-printed bionic HAp bioceramic scaffolds were observed using a scanning electron microscope (SEM, JSM-7500F, JEOL, Tokyo, Japan). Before observation, the 3D-printed HAp bioceramic scaffolds were sputter-coated with gold.

#### 2.3.2. Compressive Strength Testing

The compressive strength of the 3D-printed bionic HAp bioceramic scaffolds was measured with a crosshead speed of 0.05 mm·min^−1^ by using a universal mechanical testing machine (Instron Legend 2367 testing system, Boston, MA, USA). For each condition, 5 specimens were measured to obtain an average value.

### 2.4. In Vitro Evaluation

#### 2.4.1. In Vitro Cytocompatibility

The in vitro biocompatibility of the 3D-printed bionic HAp bioceramic scaffolds was characterized using the CCK-8 methodology. Bone marrow mesenchymal stem cells (BMSCs) cells were cultured on the 3D-printed HAp scaffolds, and the relative amounts of cell growth on these HAp scaffolds were measured after 1, 4, and 7 days. During the observation, a blank group was also set up for comparison. The absorbance was measured to determine the relative amount of cell growth. The survival of stem cells on the 3D-printed HAp scaffolds was observed and measured at 3 days of culture using a Calcein-AM/PI live/dead cell double-staining kit (YEASEN, 40747ES76, Shanghai, China). Both the live cells (yellow-green fluorescence) and the dead cells (red fluorescence) were detected simultaneously by using a fluorescence microscope (Leica, TCS SP8, Wetzlar, Germany) with a 490 ± 10 nm excitation filter.

#### 2.4.2. ALP Activity Assay

BMSCs on the 3D-printed bionic HAp bioceramic scaffolds were measured to obtain the expression levels of bone alkaline phosphatase (ALP) on days 1, 4, and 7. Purified rat ALP antibody was wrapped around a microtiter plate to make a solid-phase antibody. Firstly, ALP was added sequentially to the antibody-coated wells. Then, ALP was combined with HRP-labeled ALP antibodies to form antibody–antigen–enzyme-labeled antibody complexes. After complete scrubbing, the substrate TMB was added for color development, and TMB was converted to blue by HRP enzymes and, finally, yellow by acid. During this experiment, the cell suspension was diluted with PBS (pH 7.2–7.4) to reach a cell concentration of about 1 million/mL. Freezing and thawing were carried out repeatedly to disrupt the cells and release the intracellular components. Centrifugation was conducted for about 20 min at 2000–3000 rpm to collect the supernatant.

#### 2.4.3. Protein Expression

The protein expression was studied, including an analysis of osteopontin (OPN), runt-related transcription factor 2 (RUNX2), collagenI (Col-1), vascular endothelial growth factor 2 (VEGFR2), von Willebrand factor (vWF), and platelet endothelial cell adhesion molecule-1 (CD31) osteogenic protein expression level using the Western blot (WB) method. BMSCs were lysed and centrifuged after 21 days of osteogenic induction on the 3D-printed HAp scaffolds. The protein concentration was determined using a BCA protein concentration (Beyotime Biotechnology, Shanghai, China) assay kit.

#### 2.4.4. Statistical Analysis

For biological cell viability tests, conducting statistical analysis is crucial for enhancing the reliability, evaluating the differences, and ensuring the accuracy and scientificity of the experimental results. Therefore, all experimental results are expressed as the mean plus or minus the standard deviation (x ± s), and a chi square test, a one-way analysis of variance (oneway ANOVA), and a two-by-two comparison between sample means (q test) were performed using GraphPad Prism 9 statistical software. Statistical significance was set at a limit of *p* < 0.05. The *p*-values are displayed in the graphs. The null hypothesis that there is an association between the two was established.

## 3. Results and Discussion

### 3.1. Morphological Analysis

**Figure 3:**
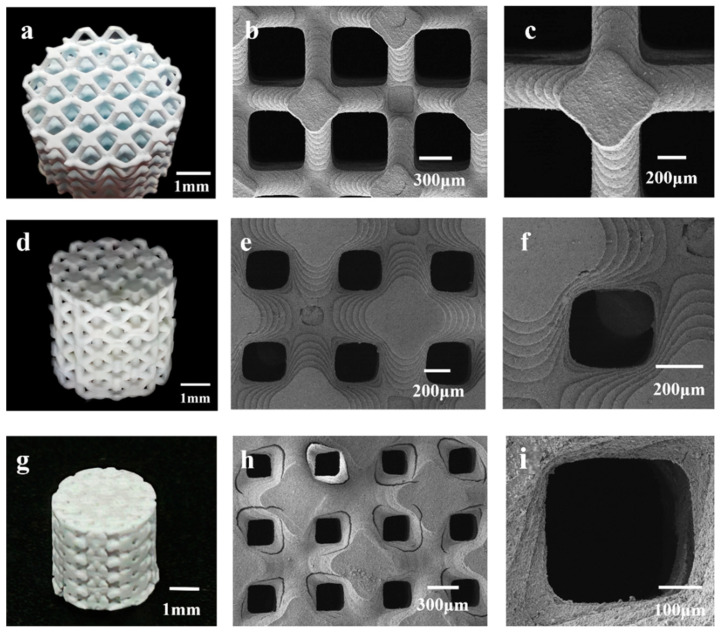
Photographs and high-magnification microstructures of 3D-printed HAp BCC scaffolds with different porosities: (**a**–**c**) B80; (**d**–**f**) B60; (**g**–**i**) B40.

Figure 3 presents a physical image and SEM images of the 3D-printed HAp BCC structures. Figure 3a shows a physical image of B80, where the average diameter is 5.38 ± 0.18 mm and the height is 5.02 ± 0.03 mm. The scaffold surface has no obvious defects, and no deformation phenomena such as bending or edge lifting have occurred. Figure 3b,c are SEM images of B80, showing clear stepped printing traces with sharp and uniform edges, and a pore size range of 400–500 μm. Figure 3d displays an image of B60, with an average diameter of 5.39 ± 0.09 mm and a height of 5.33 ± 0.07 mm. Interconnected pore structures are visible, and no deformation is observed in the scaffold. Figure 3e,f are SEM images of the scaffold, revealing pore sizes of 300–400 μm. The scaffold overall has no obvious defects and displays clear stepped traces. Figure 3g shows an image of B40, with an average diameter of 5.42 ± 0.03 mm and a height of 5.41 ± 0.04 mm. Some pores are visibly blocked, making it difficult for them to interconnect. This may be due to incomplete cleaning during the printing process, leaving some slurry inside the scaffold and causing blockage during sintering. This indicates that as porosity decreases, it becomes more difficult for internal slurry residues to flow out, coupled with shrinkage during sintering, leading to blockage. This suggests that as porosity decreases, the printing quality of the scaffold also diminishes. Figure 3h,i are SEM images of the scaffold, showing pore sizes of 200–300 μm. However, under low magnification, cracks are observed at the connections between the rods. This may be because the scaffold was removed from the muffle furnace before it had fully cooled to room temperature, resulting in excessive temperature changes and cracks. Additionally, the physical image shows pore blockage in the scaffold. When pores are in the 200–300 μm range, printing residues are more difficult to clean and remove, leading to blockage.

**Figure 4:**
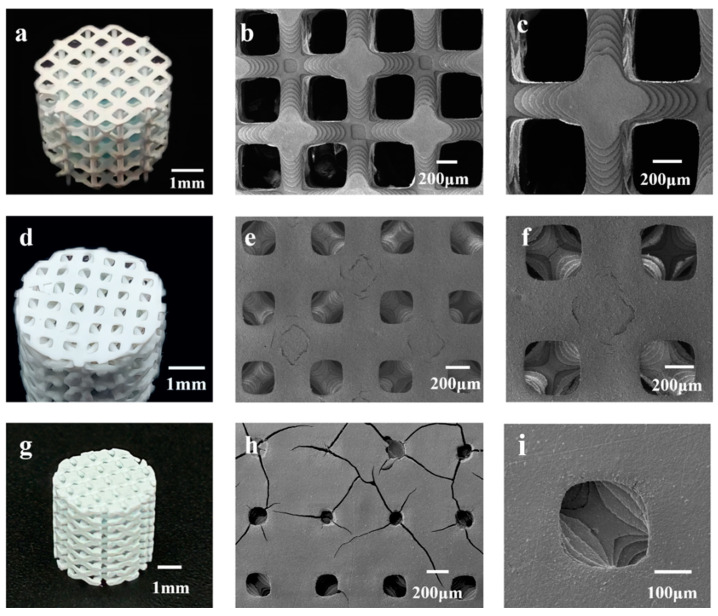
Photographs and high-magnification microstructures of 3D-printed HAp FCC scaffolds with different porosities: (**a**–**c**) F80; (**d**–**f**) F60; (**g**–i) F40.

Similarly, Figure 4 presents images of the HAp FCC scaffolds. Figure 4a shows a physical image of F80, with a diameter of 5.32 ± 0.98 mm and a height of 5.05 ± 0.21 mm. The scaffold exhibits no obvious defects such as cracks or peeling, and the pores are clear and interconnected. Figure 4b,c display the morphology of the scaffold, revealing a pore diameter range of 400–500 μm. Figure 4d is an image of F60, with a scaffold diameter of 5.41 ± 0.04 mm and a height of 5.34 ± 0.08 mm. The pores of the scaffold are clearly visible and interconnected, but there is slight curvature on the surface due to uneven shrinkage in the horizontal direction during sintering. Figure 4e,f are SEM images of F60, showing high surface quality with distinct stepped features and pore diameters ranging from 300 to 400 μm. Figure 4g shows an image of F40, with a diameter of 5.48 ± 0.03 mm and a height of 5.52 ± 0.1 mm. The scaffold surface has no obvious curvature but exhibits slight shedding. Some pores are not interconnected, which may be due to two reasons: firstly, as porosity increases and pore size decreases, incomplete cleaning after printing may lead to slurry residue blocking internal pores during sintering; secondly, during the stereolithography printing process, unstable light emission frequency may cause scattering or an inability to penetrate, resulting in uneven curing thickness and precision within the same plane. This can lead to partial pore blockage with indistinct boundaries, and after multiple cycles, these pores may be obscured and unable to interconnect. Figure 4h is an SEM image of F40, showing uneven pore sizes and obvious cracks on the surface, which may be caused by removing the scaffold from the muffle furnace before it has completely cooled, leading to a large temperature difference and surface cracking. Figure 4i is a partial enlargement of Figure 4h, showing that when the porosity is 40 vol.%, the pore size range of the HAp FCC structure is 200–300 μm.

From Figure 4, it can be concluded that for the HAp FCC structure, when the porosity is 80% and 60%, the pores within the scaffold are interconnected, achieving good molding accuracy. Specifically, when the porosity is 80%, the pore size is 400–500 μm, and when the porosity is 60 vol.%, the FCC structure has a pore size of 300–400 μm. However, when the porosity reaches 40%, the pore size is 200–300 μm, and the molding accuracy of the scaffold decreases with pore blockage, making it difficult to achieve interconnectivity. This may adversely affect subsequent in vitro and in vivo osteogenesis.

**Figure 5:**
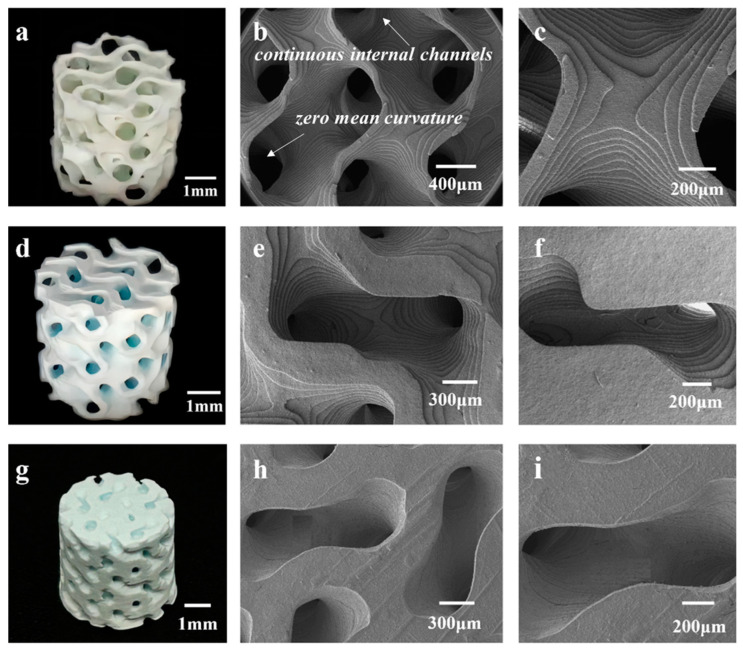
Photographs and high-magnification microstructures of 3D-printed HAp TPMS scaffolds with different porosities: (**a**–**c**) T80; (**d**–**f**) T60; (**g**–**i**) T40.

Interestingly, Figure 5 presents images of the HAp TPMS scaffolds with different porosities. Figure 5a–c and Figure 5d–f show the morphologies of TPMS structures with porosities of 80% and 60%, respectively. It can be observed that the scaffold structures are regular, with clear steps and almost no printing defects. The sintered bodies with a porosity of 80% have an average diameter of 5.85 ± 0.17 mm and a height of 5.78 ± 0.18 mm, while those with a porosity of 60 vol.% have an average diameter of 5.91 ± 0.13 mm and a height of 5.85 ± 0.11 mm. Figure 5g–i depict the T40 structure, with an average diameter of 6.16 ± 0.2 mm and a height of 6.08 ± 0.14 mm. The degree of shrinkage of the scaffold decreases as the porosity decreases. The degree of shrinkage of the TPMS structure is less than that of the BCC and FCC structures, but the shrinkage trend is the same in both the horizontal and vertical directions, with the horizontal shrinkage being less than the vertical shrinkage. This anisotropy is caused by the layer-by-layer stacking during stereolithography printing. At low magnification, the step printing traces are not visible, and the edges are relatively neat, with fewer crack defects. Compared to the BCC and FCC structures at a porosity of 40 vol.%, the printing quality of TPMS is better, with some steps still visible and no cracking. This is because the unit cell of the TPMS structure is curved, and when shrinkage occurs, the force is evenly distributed between each surface. In contrast, the BCC and FCC structures are composed of rods and nodes, and when shrinkage occurs, the force is unevenly distributed, leading to curling and edge lifting.

It should also be noted that some surface cracks and uneven pore sizes were detected in some scaffolds, as shown in Figure 3, Figure 4 and Figure 5. Cracks and defects always happen among 3D-printed ceramics and their structures. The reasons for these cracks and defects (usually pores, stratification, and so on) are complicated, usually including the slurry’s stability and homogeneous state, 3D printing parameters, debinding and sintering parameters, and residual thermal stress, and so on [39,40]. Determining how to eliminate or reduce these defects and cracks is a complex, difficult, systematic, and long-term technological challenge. It is believed that even with pores, which are inherently caused by the process and have a significant impact on the subsequent mechanical properties, such small-scale cracks and pores (compared to the structure’s porosity) have little impact on biological performance. Therefore, we believe that this work focuses on the impact patterns of porous design on mechanical and biological properties, without delving deeply into the effects of internal defects and cracks in 3D-printed ceramic materials

### 3.2. Compressive Strength

**Figure 6:**
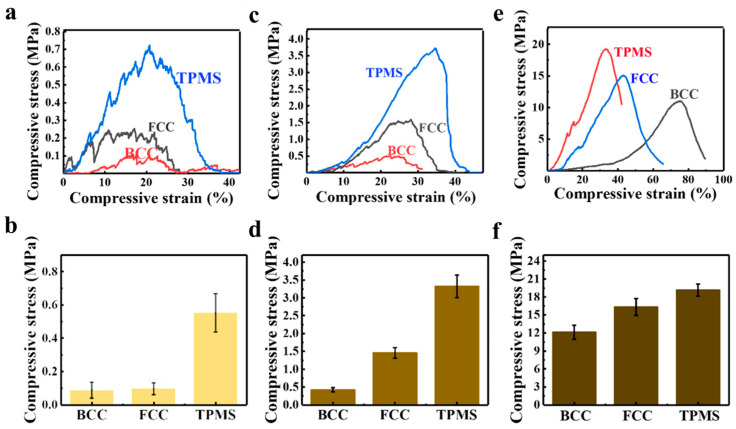
Compressive strength of 3D-printed HAp scaffolds with different porosities: (**a**,**b**) 80 vol.%; (**c**,**d**) 60 vol.%; (**e**,**f**) 40 vol.%. These graphs show that TPMS structures have the highest compressive strength.

The compressive strength of the 3D-printed bionic HAp bioceramic scaffolds were tested. Table 1 gives the compressive strength of various HAp scaffolds in this study, and Figure 6 presents all the compressive strength testing cures of the HAp scaffolds. As listed in Table 1, when the porosity is 80 vol.%, the mechanical properties of the B80, F80, and T80 structures are 0.14 ± 0.04, 0.088 ± 0.05, and 0.50 ± 0.12 MPa, respectively. The stress–strain curves in Figure 6a,b show the irregularities and burrs, which are due to the small size and thin diameter of the rods, making them prone to fracture during compression, resulting in burrs. When the porosity is 60 vol.%, the mechanical properties of the B60, F60, and T60 structures are 1.59 ± 0.15, 1.81 ± 0.27, and 3.32 ± 0.32 MPa, respectively. At this point, the stress–strain curves are smoother (as shown in Figure 6c,d), especially for the TPMS structure. When the porosity is 40 vol%, the mechanical properties of the B40, F40, and T40 structures are 12.12 ± 1.17, 16.34 ± 1.40, and 19.15 ± 1.02 MPa, respectively.

It is clearly that when the porosity is constant, the mechanical properties of the BCC, FCC, and TPMS structures tend to increase sequentially. Figure 6b,d,f presents compressive strength bar charts for the BCC, FCC, and TPMS structures. It is found that, regardless of porosity, the TPMS structure consistently exhibits the best mechanical properties, while the BCC structure performs the worst. This is because the structural design of TPMS achieves stress shielding, avoiding stress concentration and thus demonstrating better compressive strength. However, in the BCC and FCC structures, the connections between rods are most susceptible to becoming stress concentration points, leading to fracture when subjected to force. In one word, TPMS scaffolds exhibit the best compressive strength. As reported previously [41,42], the reason that TPMS structures have better mechanical properties can be answered as follows: Firstly, TPMS structures consist of continuous and smooth surfaces, which have a large internal surface area and continuous internal channels, causing less stress concentration. This continuity and smoothness help to disperse and absorb external loads, thereby enhancing the overall integrity and stability of the structure. Secondly, the surface morphology of the TPMS structure has zero mean curvature, which effectively reduces the local stress concentration. This characteristic allows the TPMS structure to distribute stress more evenly under load, reducing the risk of damage caused by stress concentration, thereby providing superior mechanical performance. Finally, the stress distribution in TPMS structures under compression is relatively uniform, which helps to achieve a stable collapse mechanism and energy absorption performance. Uniform stress distribution means that the structure can absorb and disperse energy more evenly when compressed, thereby enhancing its energy absorption capacity during loading. In our experiments and numerical simulations, the TPMS structures show relatively uniform stress distribution across all unit cells, which helps to reduce local damage to the structure when subjected to loading, and improves the overall stability and safety of the structure.

In addition, it is also evident that porosity has a significant impact on the compressive strength of scaffolds. At a porosity of 80 vol.%, the scaffolds have compressive strength ranging from 0.09 to 0.55 MPa. When the porosity is reduced to 60 vol.%, the compressive strengths of all three structural types increase 4 to 5 times compared to those at 80% porosity. When the porosity further decreases to 40 vol.%, the highest compressive strength, which is in the TPMS structure, can reach 19.15 MPa, representing an increase of approximately 35 times compared to the TPMS at 80% porosity and about 6 times compared to that at 60% porosity.

### 3.3. Biological Safety Assessment

The survival and proliferation of cells on HAp scaffolds are important evaluation indicators for assessing their safety. We used a CCK-8 kit to monitor cell proliferation and viability on scaffolds on days 1, 4, and 7. A blank control group without a scaffold was used for comparison. By establishing a fitting relationship between cell number and OD value, we evaluated the biological safety and cytocompatibility of the HA scaffolds.

**Figure 7:**
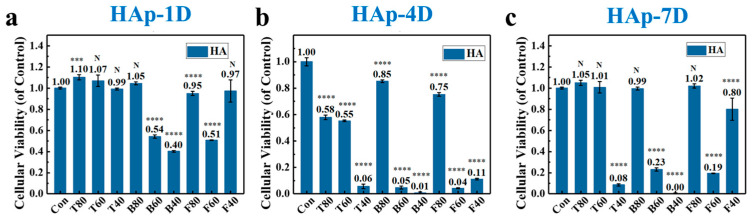
CCK-8 cell viability assay for 3D-printed HAp scaffolds with different structures and porosities: (**a**) 1 day; (**b**) 4 days; (**c**) 7 days. (Note: asterisk indicates a statistically significant difference between the two groups; N: no statistically significant difference; ***: *p* < 0.001; ****: *p* < 0.0001.)

The results of culturing cells on 3D-printed HAp scaffolds for 1 day are shown in Figure 7a. It can be observed that the growth on T80 is better compared to the blank control group (*p* < 0.05), with a statistically significant difference. The T60, T40, B80, and F40 scaffolds also provide conditions supportive of cell adhesion and growth, but there are no statistically significant differences in the proliferation results compared to the blank group. However, the B60, B40, F80, and F60 scaffolds do not support cell proliferation or even growth compared to the blank group, exhibiting some toxicity. These four scaffolds show statistically significant differences compared to the blank control group (*p* < 0.01). After 4 days of cell culture (as shown in Figure 7b), the T80, T60, B80, and F80 scaffolds exhibit some cytotoxicity, with a relatively lower number of cells compared to the blank group. For the other five scaffolds, almost no cell survival is observed compared to the blank group, indicating that the scaffold materials inhibit cell growth to some extent within the first 4 days. All results show statistically significant differences (*p* < 0.01). When cells are cultured for 7 days, the number of cells on the T80, T60, B80, and F80 scaffolds increases significantly compared to day 4, and there are no significant differences compared to the blank group on day 7, indicating that the presence of the scaffolds does not significantly promote cell proliferation. The F40 scaffold also shows an increase compared to day 4, but it does not reach the number of cells in the blank group on day 7, with statistically significant differences compared to the blank control group (*p* < 0.01). The relative number of cells on the remaining T40, B60, and F60 scaffolds remains low, with statistically significant differences observed (*p* < 0.01). This suggests that HAp scaffolds have biocompatibility but have a weak effect on cell proliferation, and the mild cytotoxicity observed on day 4 may be due to contamination of the culture environment.

Based on these experimental results, it is found that scaffolds with porosity of 80 vol.% and 60 vol.% were more likely to support cell growth, while it is difficult for cell numbers to reach levels comparable to the blank control group when the porosity is 40 vol.%. This may be due to the interconnected pores within the structure of scaffolds with high porosity, which provides a favorable environment for cell adhesion and expansion. Additionally, the mechanical properties of scaffolds with porosity of 80 vol.% and 60 vol.% can meet the requirements of non-load-bearing areas such as cartilage and jaw bones. These scaffolds have pores that are interconnected vertically and horizontally, offering potential to support cell proliferation, adhesion, and expansion. In contrast, according to the SEM images, scaffolds with a porosity of 40 vol.% have smaller pore sizes of 200~300 μm on their surfaces. Due to the lower porosity, the internal pores cannot achieve complete interconnection and communication, and even the peripheral pores may be closed. This directly hinders cell adhesion and may potentially have adverse effects on subsequent osteogenic behavior in vivo. Therefore, we only chose the HAp scaffolds with porosity of 80 vol.% and 60 vol.% in the following investigations.

**Figure 8:**
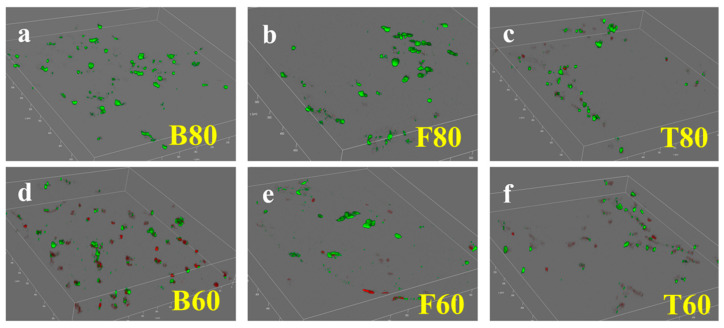
Fluorescence images of live/dead cell staining on various 3D-printed HAp scaffolds: (**a**) B80; (**b**) F80; (**c**) T80; (**d**) B60; (**e**) F60; (**f**) T60.

Figure 8 shows fluorescence images of live/dead cell staining on various HAp scaffolds. In Figure 8a–c, which depict the BCC, FCC, and TPMS structures with a porosity of 80 vol.%, respectively, there is little to no visible distribution of dead cells, and live cells stained with green fluorescence occupy a large area. This is consistent with the results of the CCK-8 cytotoxicity test. In contrast, for the BCC, FCC, and TPMS structures with a porosity of 60 vol.% (as given in Figure 8d–f), dead cells stained with red fluorescence can be clearly observed, which aligns with the CCK-8 results. This indicates that lower porosity of the scaffolds inhibits cell growth and is not conducive to cell expansion and proliferation. Additionally, smaller pore sizes hinder the inflow of nutrient-laden extract solutions required for cell survival, leading to cell death. The live/dead cell staining results demonstrate that HAp ceramic possesses good biocompatibility.

### 3.4. In Vitro Observation of Osteogenesis on Implants

**Figure 9:**
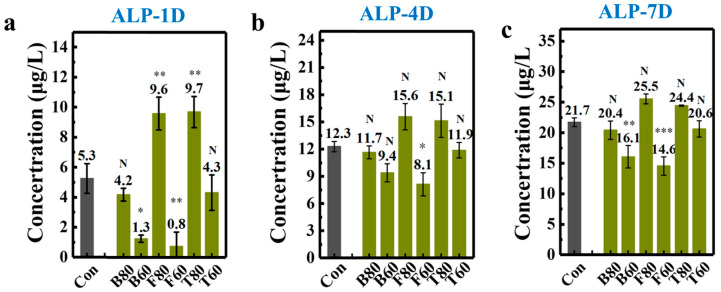
Expression of cellular alkaline phosphatase activity on days (**a**) 1, (**b**) 4, and (**c**) 7 (Note: asterisk indicates statistical differences between the two groups; N: no statistically significant difference; *: *p* < 0.05; **: *p* < 0.01; ***: *p* < 0.001).

The activity of alkaline phosphatase (ALP) is an important indicator of early osteogenic differentiation, and ALP serves as a crucial marker for cellular osteogenic differentiation. To analyze and evaluate the impact on cellular osteogenic differentiation behavior, we examined the expression of ALP activity in BMSCs cultured on the 3D-printed HAp scaffolds after 1, 4, and 7 days. The results of the assay are shown in Figure 9.

Figure 9a presents the ALP expression levels of HAp scaffolds after 1 day of culture. The green and blue bars represent HA scaffolds with F80 and T80 structures showing higher expression levels (9.59 ± 1.08 and 9.69 ± 1.04 μg/L, respectively) (*p* < 0.01, statistically significant differences). The ALP expression of B80 and T60 structures is not statistically significantly different from that in the control group, while the ALP expression of B60 and F60 structures is lower, with statistically significant differences (*p* < 0.05). After 4 days of culture, as shown in Figure 9b, the F80 and T80 structures still maintain higher levels, with 15.59 ± 1.46 and 15.12 ± 1.84 μg/L, respectively. The B80 and T60 structures still maintain expression levels that are not statistically different from those in the control group, which is similar to the trend observed on the first day. The ALP levels of the B60 and F60 structures are still slightly lower than those in the control group, with statistically significant differences for the F60 structure. According to the ALP expression levels on the fourth day, the expression of ALP enzymes in all 12 scaffolds continues to increase, consistent with the trend observed on the first day. Figure 9c shows the expression results of HA scaffolds after 7 days of cell culture on the scaffolds. Among them, the F80 and T80 structures (25.52 ± 0.80 and 24.42 ± 0.09 μg/L, respectively) continue to maintain the highest levels. B60 and F60 are still slightly lower than in the control group (*p* < 0.05, statistically significant differences). The growth trend of all scaffolds is the same as that observed on the first and fourth days, indicating that the cell culture environment was relatively stable and the data are reliable. It should be noted that the reason for the mild cytotoxicity on day 4 is not clear enough, and should be studied in detail in future work.

How porosity and structural design affect early osteogenic differentiation is very interesting. On the one hand, porosity is one of the important factors affecting cell proliferation and differentiation. Studies have shown that the porosity and pore size of porous materials can be adjusted as needed, and even interconnected porous structures can be obtained, making them ideal substitutes for bone tissue. Appropriate porosity can provide enough space for cell growth and proliferation while promoting the transport of nutrients and the expulsion of metabolic waste, which is crucial for cell activity and differentiation. One the other hand, the pore structure of porous materials has a direct impact on cell activity. For example, studies on the biocompatibility of porous HAp ceramics have shown that porous HAp ceramic materials are non-toxic to cells and have no potential for skin sensitization, exhibiting good biocompatibility [25]. Moreover, they have shown similar effects to hydroxyapatite in repairing bone defects, indicating their good osteoconductivity. This suggests that the porous structure can promote cell adhesion and growth on the material surface, thereby enhancing ALP activity.

**Figure 10:**
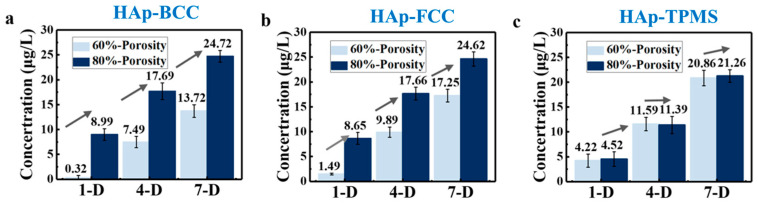
Effects of porosity on cellular alkaline phosphatase activity of 3D-printed HAp scaffolds.

Furthermore, in order to facilitate the analysis of the effect of porosity on the expression of ALP (alkaline phosphatase) enzyme activity on scaffolds, the aforementioned data were classified and statistically analyzed. Figure 10 visually represents the ALP expression on scaffolds with porosities of 80 vol.% and 60 vol.% on days 1, 4, and 7. It can be observed that the expression level at 80 vol.% porosity is significantly higher than that at 60 vol.% porosity, with the experimental results for BCC, FCC, and TPMS structures supporting this conclusion. This is because when the porosity is 80 vol.%, the interconnected pores within the scaffold allow nutrients and moisture to more easily enter the interior, facilitating cell growth and expansion, and thus promoting the differentiation and expression of osteoblasts. It also indicates that porosity has a greater impact on osteogenic differentiation than component and structure. Therefore, exploring the optimal size of scaffolds in bone repair engineering is of significant research importance. Furthermore, scaffolds with pore sizes of 400–500 μm demonstrate greater potential for bone repair than those with pore sizes of 300–400 μm. The previous CCK-8 experimental results also support this conclusion.

### 3.5. Biomedical Application Potential Analysis

Based on the above conclusions, the HAp scaffold with a porosity of 80 vol.% and a TPMS structure demonstrates great potential for bone repair. The HAp material has good biocompatibility and osteogenic induction ability. This structure provides pores that can support cell expansion and adhesion, creating a favorable environment for cell growth and osteogenic induction. It has the potential to support bone regeneration and reconstruction in vivo. Compared with Chen’s work [23], the effects of structural configuration and porosity were discussed in depth in our work. Three different structures, including FCC, BCC, and TPMS, were designed, and three different porosities, 80 vol.%, 60 vol.%, and 40 vol.%, were chosen. However, Chen et al. implanted the HAp structure into rabbits and conducted a systematic study, and we should conduct similar studies in the future. In addition, compared with Luo’s work [24], the accuracy of VPP 3D printing in this work was much higher than that of DIW.

In addition, it should be noted that the presence of non-interconnected pores is concerning for nutrient flow and cell migration, critical aspects for bone regeneration. In this study, the state of pores is strongly related to the bionic design. Different structures (body-centered cubic (BCC), face-centered cubic (FCC), and gyroid Triply Periodic Minimal Surfaces (TPMSs)) and porosities (80 vol.%, 60 vol.%, and 40 vol.%) were chosen and designed, resulting in certainty regarding the state of the pores. In our future work, we will design more structures which consider the presence of non-interconnected pores, and study their effects on the following nutrient flow and cell migration, critical aspects for bone regeneration.

In summary, the aim of this study is to find the optimal structure and porosity for HAp bioceramic scaffolds. In this study, the effects of structure and porosity on the morphology, compressive strength, and in vitro behaviors of the HAp scaffolds were studied and compared with each other. We want to provide a reference for the application and development of HAp scaffolds in the field of bone repair engineering.

## 4. Conclusions

Herein, bionic hydroxyapatite (HAp) ceramic scaffolds with different structures and porosities were designed, 3D printed, and characterized. The effects of structure and porosity on the morphology, compressive strength, and in vitro behaviors of the 3D-printed HAp scaffolds were studied. The main conclusions are as follows:

(1) The porosity has a significant impact on the pore diameter and surface morphology of the three structures: BCC, FCC, and TPMS. Structures of these three types with porosities of 80 vol.%, 60 vol.%, and 40 vol.% are successfully bionically designed and 3D printed. When the porosity is higher, the pore diameters of the FCC and BCC structures range from 400 to 500 μm, and their surface morphologies are better. At a porosity of 60 vol.%, the pore sizes of FCC and BCC are 300–400 μm, with good print quality, clear boundaries, and visible step-like printing traces. However, when the porosity is lower at 40 vol.%, the pore sizes of the FCC and BCC structures are 200–300 μm, and a small number of pores are observed to be blocked. There are slight cracks on the surface of the scaffolds, and the surface quality of the TPMS structure decreases slightly, but no cracking occurs.
(2) Porosity has a significant impact on the compressive strength of 3D-printed HAp scaffolds. Among the three structures of FCC, BCC, and TPMS, the TPMS structure exhibits the best mechanical properties, while the BCC structure has the worst. At a porosity of 80 vol.%, the compressive strength of the TPMS structure is 5–6 times that of the BCC structure. When the porosity is 60 vol.%, the TPMS is 6–7 times stronger than that of BCC. At a porosity of 40 vol.%, the compressive strength of the TPMS is approximately 1.7 times that of BCC.
(3) All three structures, FCC, BCC, and TPMS, have the ability to promote cell proliferation, with the TPMS structure demonstrating better support for cell survival. All three structures also exhibit early osteogenic differentiation capabilities and the ability to promote the expression of osteogenic proteins.
(4) Porosities of 80 vol.% and 60 vol.% have a significant impact on in vitro biological performance: Compared to a porosity of 60 vol.%, scaffolds with a porosity of 80% show better cell growth and proliferation, and are more capable of promoting the early osteogenic differentiation of cells. They also have a significant ability to induce the expression of osteogenic proteins and have greater potential as scaffolds for repairing bone defects.

Based on the above conclusions, the HAp scaffold with a porosity of 80 vol.% and a TPMS structure demonstrates great potential for bone repair. The HAp material has good biocompatibility and osteogenic induction ability. This structure provides pores that can support cell expansion and adhesion, creating a favorable environment for cell growth and osteogenic induction. It has the potential to support bone regeneration and reconstruction in vivo.

## Figures and Tables

**Table 1:** Compressive strength of various 3D-printed HAp scaffolds.

Structure/Porosity	Compressive Strength (MPa)		
	80 vol.%	60 vol.%	40 vol.%
BCC	0.14 ± 0.04	1.59 ± 0.15	12.12 ± 1.17
FCC	0.088 ± 0.05	1.81 ± 0.27	16.34 ± 1.40
TPMS	0.50 ± 0.12	3.32 ± 0.32	19.15 ± 1.02

## Data Availability

The original contributions presented in this study are included in the article. Further inquiries can be directed to the corresponding author.

## References

[1] Alonzo A., Primo F.A., Kumar S.A., Mudloff J.A., Dominguez E., Fregoso G., Oritz N., Weiss W.M., Jaddar B. (2021). Bone tissue engineering techniques, advances, and scaffolds for treatment of bone defects. Curr. Opin. Biomed. Eng..

[2] Li J., Han F., Ma J., Wang H., Pan J., Yang G., Zhao H., Zhao J., Liu J., Liu Z. (2022). Targeting endogenous hydrogen peroxide at bone defects promotes bone repair. Adv. Funct. Mater..

[3] Wu J., Zhang Y., Lyu Y., Cheng L. (2023). On the various numerical techniques for the optimization of bone scaffold. Materials.

[4] Shi J., Dai W., Gupta A., Zhang B., Wu Z., Zhang Y., Pan L., Wang L. (2022). Frontiers of hydroxyapatite cmposites in bonic bne tssue egineering. Materials.

[5] Lv X., Wang S., Xu Z., Liu X., Liu G., Cao F., Ma Y. (2023). Structural mehanical poperties of 3D pinting bomimetic bone replacement materials. Biomimetics.

[6] Ghayor C., Weber F.E. (2018). Osteoconductive microarchitecture of bone substitutes for bone regeneration revisited. Front. Physiol..

[7] Carluccio D., Demir A.G., Bermingham M.J., Dargusch M.S. (2020). Challenges and opportunities in the selective laser melting of biodegradable metals for load-bearing bone scaffold applications. Metall. Mater. Trans. A.

[8] Goswami M., Rekhi P., Debnath M., Ramakrishna S. (2021). Microbial polyhydroxyalkanoates granules: An approach targeting biopolymer for medical applications and developing bone scaffolds. Molecules.

[9] Miri Z., Haugen H.J., Loca D., Rossi F., Perale G., Mohjanian A., Ma Q. (2024). Review on the strategies to improve the mechanical strength of highly porous bone bioceramic scaffolds. J. Eur. Ceram. Soc..

[10] Khalaf A.T., Wei Y., Wan J., Zhu J., Peng Y., Kadir S.Y.A., Zainol J., Oglah Z., Cheng L., Shi Z. (2022). Bone tissue engineering through 3D bioprinting of biocramic scaffolds: A review and update. Life.

[11] Mahmound E.M., Sayed M., El-Kady A.M., Elasayed H., Naga S.M. (2020). n vitro and in vivo study of naturally derived alginate/hydroxyapatite bio composite scaffolds. Int. J. Biol. Macromol..

[12] Wang Q., Ma Z., Wang Y., Zhong L., Xie W. (2021). Fabrication and characterization of 3D printed biocomposite scaffolds based on PCL and zirconia nanoparticles. Bio-Des. Manuf..

[13] Radulescu D.E., Vasile O.R., Andronescu E., Ficai A. (2023). Latest research of doped hydroxyapatite for bone tissue engineering. Int. J. Mol. Sci..

[14] Shreya R., Fopase R., Sharama S., Pandey L.M. (2024). Design of biphasic Fe and Zn doped hydroxyapatite: Novel strategy for combating osteomyelitis infections. Ceram. Inter..

[15] He H., Wang L., Cai X., Wang Q., Liu P., Xiao J. (2023). Biomimetic collagen composite matrix-hydroxyapatite scaffold induce bone regeneration in critical size cranial defects. Mater. Design.

[16] Feng C., Zhang K., He R., Ding G., Xia M., Jin X., Xie C. (2020). Additive manufacturing of hydroxyapatite bioceramic scaffolds: Dispersion, digital light processing, sintering, mechanical properties, and biocompatibility. J. Adv. Ceram..

[17] Shan Y., Bai Y., Yang S., Zhou Q., Wang G., Zhu B., Zhou Y., Fang W., Wen N., He R. (2023). 3D-printed strontium-incorporated β-TCP bioceramic triply periodic minimal surface scaffolds with simultaneous high porosity, enhanced strength, and excellent bioactivity. J. Adv. Ceram..

[18] Wong S.K., Yee M.M.F., Chin K.Y., Ima-Nirwana S. (2023). A review of the application of natural and synthetic scaffolds in bone regeneration. J. Funct. Biomater..

[19] Gupta K., Meena K. (2023). Artificial bone scaffolds and bone joints by additive manufacturing: A review. Bioprint.

[20] Sopyan I., Toibah A.R., Ramesh S., Mel E., Algap A.S.F. (2024). Magnesium-enhanced porous hydroxyapatite ceramics: Morphometric parameters, physical properties and bioactivity. Ceram. Int..

[21] Liu R., Ma L., Liu H., Xu B., Feng C., He R. (2021). Effects of pore size on the mechanical and biological properties of stereolithographic 3D printed HAp bioceramic scaffold. Ceram. Int..

[22] Chen Q., Zou B., Lai Q., Wang Y., Xue R., Xing H., Fu X., Huang C., Yao P. (2019). A study on biosafety of HAP ceramic prepared by SLA-3D printing technology directly. J. Mech. Behav. Biomed. Mater..

[23] Alexander A.E., Wake N., Chepelev L., Brantner P., Ryan J., Wang K.C. (2021). A guideline for 3D printing terminology in biomedical research utilizing ISO/ASTM standards. 3D Print. Med..

[24] Luo Y., Zhai D., Huan Z., Zhu H., Xia L., Chang J., Wu C. (2015). Three-dmensional pinting of hllow-sruts-pcked boceramic saffolds for bne rgeneration. ACS Appl. Mater. Interface.

[25] Liu K., Zhou Q., Zhang X., Ma L., Xu B., He R. (2023). Morphologies, mechanical and in vitro behaviors of DLP-based 3D printed HA scaffolds with different structural configurations. RSC Adv..

[26] Ma H., Feng C., Chang J., Wu C. (2018). 3D-printed bioceramic scaffolds: From bone tissue engineering to tumor therapy. Acta Biomater..

[27] Lin H., Zhang L., Zhang Q., Wang Q., Wang X., Yan G. (2023). Mechanism and application of 3D-printed degradable bioceramic scaffolds for bone repair. Biomater. Sci..

[28] Zhang H., Fuh L.J., Hsu J.T., Lim Z.M., Huang H.L. (2024). Biomechanical analysis of partial mandibular implants with various lattice designs of different material properties: In vitro study and finite element analysis. Int. J. Bioprint..

[29] Ngo T.D., Kahsa A., Imbalzano G., Nguyen K.T.Q., Hui D. (2018). Additive manufacturing (3D printing): A review of materials, methods, applications and challenges. Compos. B Eng..

[30] Parisien A., ElSayed M.S.A., Frei H. (2022). Mechanoregulation modelling of stretching versus bending dominated periodic cellular solids. Mater. Today Commun..

[31] Wang J., Tang Y., Cao Q., Wu Y., Wang Y., Yuan B., Li X., Zhou Y., Chen X., Zhu X. (2022). Fabrication and biological evaluation of 3D-printed calcium phosphate ceramic scaffolds with distinct macroporous geometries through digital light processing technology. Regen. Mater..

[32] Liang H., Wang Y., Cheng S., Liu Y., Liu Z., Bai J. (2022). Nano-hydroxyapatite bone scaffolds with different porous structures processed by digital light processing 3D printing. Int. J. Bioprint..

[33] Dong Z., Zhao X. (2021). Application of TPMS structure in bone regeneration. Eng. Regen..

[34] Yu S., Sun J., Bai J. (2019). Investigation of functionally graded TPMS structures fabricated by additive manufacturing. Mater. Des..

[35] Maevskaia E., Guerrero J., Ghayor C., Bhattacharya I., Weber F.E. (2023). TPMS-based scaffolds for bone tissue engineering: A mechenical, in vitro and in vivo study. Tissue Eng. Part A.

[36] Bobbert F.S.L., Lietaert K., Eftekhari A.A., Pouran B., Ahmadi S.M., Weinans H., Zadpoor A.A. (2017). Additively manufactured metallic porous biomaterials based on minimal surfaces: A unique combination of topological, mechanical, and mass transport properties. Acta Biomater..

[37] Zhou Q., Su X., Wu J., Zhang X., Su R., Ma L., Sun Q., He R. (2023). Additive manufacturing of bioceramic implants for restoration bone engineering: Technologies, advances, and future perspectives. ACS Biomater. Sci. Eng..

[38] Li S., Shan Y., Chen J., Chen X., Shi Z., Zhao L., He R., Li Y. (2024). 3D printing and biomedical applications of piezoelectric composites: A critical review. Adv. Mater. Technol..

[39] Zhang K., Meng Q., Qu Z., He R. (2023). A review of defects in vat photopolymerization additive-manufactured ceramics: Characterization, Control, and Challenges. J. Eur. Ceram. Soc..

[40] Zhang K., Meng Q., Zhang X., Qu Z., He R. (2022). Quantitative characterization of defects in stereolithographic additive manufactured ceramic using X-ray computed tomography. J. Mater. Sci. Technol..

[41] Lu J., Zhang X., Li S., Zhang L., Wang W., Li Z., Zhang Y., Wang G., Li Y., He R. (2023). Quasi-static compressive and cyclic dynamic impact performances of vat photopolymerization 3D printed Al_2_O_3_ triply periodic minimal surface scaffolds and Al_2_O_3_/Al hybrid structures: Effects of cell size. J. Alloys Compd..

[42] Zhang X., Su R., Gao X., Chen J., Liu G., He R., Li Y. (2024). Circumventing the brittleness of 3D-printed Al2O3 cellular ceramic structures via compositing with polyurea. Rare Met..

